# Poly(4-hydroxybutyrate) (P4HB) production in recombinant *Escherichia coli*: P4HB synthesis is uncoupled with cell growth

**DOI:** 10.1186/1475-2859-12-123

**Published:** 2013-12-11

**Authors:** Sylvaine Le Meur, Manfred Zinn, Thomas Egli, Linda Thöny-Meyer, Qun Ren

**Affiliations:** 1 Laboratory for Biomaterials, Swiss Federal Laboratories for Materials Science and Technology (Empa), Lerchenfeldstrasse 5, CH-9014, St. Gallen, Switzerland; 2 Swiss Federal Institute of Technology Zurich (ETH), Environmental Sciences, Rämistrasse 101, 8092, Zurich, Switzerland; 3 Biotechnology, HES-SO Valais Wallis, Rue du Rawyl 64, P.O.B. 2134, CH-1950 Sion, Switzerland; 4 Environmental Microbiology, Swiss Federal Institute of Aquatic Science and Technology (Eawag), Überlandstrasse 133, P.O. Box 610, CH-8600, Dübendorf, Switzerland

**Keywords:** P4HB, Xylose, Bioprocess development, Batch culture

## Abstract

**Background:**

Poly(4-hydroxybutyrate) (P4HB), belonging to the family of bacterial polyhydroxyalkanoates (PHAs), is a strong, flexible and absorbable material which has a large variety of medical applications like tissue engineering and drug delivery. For efficient production of P4HB recombinant *Escherichia coli* has been employed. It was previously found that the P4HB synthesis is co-related with the cell growth. In this study, we aimed to investigate the physiology of P4HB synthesis, and to reduce the total production cost by using cheap and widely available xylose as the growth substrate and sodium 4-hydroxybutyrate (Na-4HB) as the precursor for P4HB synthesis.

**Results:**

Six different *E. coli* strains which are able to utilize xylose as carbon source were compared for their ability to accumulate P4HB. *E. coli* JM109 was found to be the best strain regarding the specific growth rate and the P4HB content. The effect of growth conditions such as temperature and physiological stage of Na-4HB addition on P4HB synthesis was also studied in *E. coli* JM109 recombinant in batch culture. Under the tested conditions, a cellular P4HB content in the range of 58 to 70% (w w^-1^) and P4HB concentrations in the range of 2.76 to 4.33 g L^-1^ were obtained with a conversion yield (Y_P4HB/Na-4HB_) of 92% w w^-1^ in single stage batch cultures. Interestingly, three phases were identified during P4HB production: the “growth phase”, in which the cells grew exponentially, the “accumulation phase”, in which the exponential cell growth stopped while P4HB was accumulated exponentially, and the “stagnation phase”, in which the P4HB accumulation stopped and the total biomass remained constant.

**Conclusions:**

P4HB synthesis was found to be separated from the cell growth, i.e. P4HB synthesis mainly took place after the end of the exponential cell growth. High conversion rate and P4HB contents from xylose and precursor were achieved here by simple batch culture, which was only possible previously through fed-batch high cell density cultures with glucose.

## Background

Natural polyhydroxyalkanoates (PHAs) are synthesized by many microorganisms as carbon and energy storage compounds and deposited as granules in their cytoplasm. PHA accumulation appears when bacterial cells grow under conditions where nutrients other than carbon source, such as nitrogen or phosphorus, are limiting growth. Depending on the carbon substrate supplied, PHAs with different composition are produced. They are classified as short-chain, medium-chain and long-chain length PHAs according to the number of carbon atoms of the monomeric units [1]. Over a hundred different carboxylic acid monomers were reported to be incorporated into PHAs, resulting in polymers with a wide range of material properties [1]. These natural polymers have attracted particular attention due to their biodegradability and biocompatibility [2]–[4]. Among them, poly(4-hydroxybutyrate) (P4HB) is a highly interesting polymer for various biomedical applications [5].

P4HB biosynthesis has been studied for about 20 years and it was, and still is, the first and only PHA-based product approved by the FDA as an absorbable suture for clinical application. It is a strong, flexible thermoplastic material that can be processed easily to scaffolds, heart valves or cardiovascular tissue supports [5]. The most remarkable property of P4HB is its very high elasticity and molecular weight, as both benchmark closely to ultra-high molecular weight polyethylene [6]; it can be stretched 10-times its original length before breaking [5]. In addition, P4HB is biocompatible and extremely well tolerated *in vivo* because biological hydrolysis of P4HB yields 4HB, which is a common metabolite in the human body [7]. When used *in vivo*, the degradation of P4HB implant takes place *via* surface erosion and does not lead to a burst release of acid, which is an immense advantage for medical applications [5]. Thus, it is highly desired to obtain P4HB in large scale at a competitive cost. It was reported that up to 50% of the total cost of poly(3-hydroxybutyrate) (P3HB) arises from the carbon source [8]. Therefore, to reduce the cost of the carbon source used for large scale P4HB production, agricultural derived feedstock such as processed hemicelluloses may be employed as a co-substrate to produce the bacterial biomass.

Annually, 60 billion tons of hemicelluloses are produced and remain mostly unused [9]. Hemicellulose is the third most abundant polymer in nature and can be hydrolyzed into simple sugars by either chemical or enzymatic hydrolysis [10]. The dominant building unit of hemicelluloses is xylose. In some plants, xylose polymer (xylan) comprises up to 40% of the total dry plant material. Xylose can be used as an industrially relevant carbon source for bacterial growth, for example, by *Escherichia coli* strains [11].

Up to now, several wild-type bacterial strains have been reported to be able to produce P(3HB-*co*-4HB) copolymer: *Ralstonia eutropha*, *Alcaligenes latus*, *Comamonas acidovorans, Comamonas testosteroni* and *Hydrogenophaga pseudoflava*[12,13]. Saito and coworkers reported the production of P(3HB-*co*-4HB) copolymers by *R. eutropha* using different carbon sources with or without 4HB as precursor, however, only very low cellular polymer contents were obtained [13]. It was also reported that a maximum of 21% w w^-1^ of P4HB can be achieved by *C. acidovorans* when using 4HB or 1,4-butanediol as precursor [13]. Kim and colleagues performed fed-batch experiments with *R. eutropha* supplying in the first step fructose and in the second step only 4HB. They obtained a cell concentration of 33.6 g L^-1^ and a P(3HB-*co*-4HB) copolymer content of 41.7% w w^-1^ with 25 mol% 4HB [12]. To produce P4HB homopolymers recombinant strains were mainly used.

It has been shown previously that microorganisms that do not produce PHA naturally are ideally suited for the manipulation of the levels of the PHA biosynthetic enzymes and, hence, allow to increase polymer productivity [14]. Wild-type *E. coli* strains cannot synthesize any type of PHA, including P4HB. By introducing the P4HB synthesizing genes, recombinant *E. coli* strains are able to produce P4HB through the newly acquired biosynthetic pathway. It has been reported that the overexpression of PHA synthase (*phaC*) and β-ketothiolase (*phaA*) genes from *R. eutropha* allowed *C. acidovarans* to produce up to 51% w w^-1^ P4HB [15]. By introducing *phaC* from *R. eutropha* and a 4-hydroxybutyric acid-coenzyme A transferase gene (*orfZ*) from *Clostridium kluyveri, E. coli* strain XL1-Blue was able to produce P4HB when 4HB was supplied as a precursor in the culture medium [16]. A P4HB content of 58.5% w w^-1^ was achieved in 100 mL shake flasks, however, information on the biomass concentration was not mentioned [16]. Recently, Zhou et al. reported that *E. coli* JM109 mutant carrying two plasmids reached about 1.9 g L^-1^ P4HB and 35% (w w^-1^) P4HB using LB medium containing glucose in a batch culture [17]. There, LB rich medium was applied and two antibiotics were needed to keep the plasmids, which might be too expensive for large-scale production.

The importance of choosing a suitable *E. coli* host strain for recombinant culture cultivation was demonstrated by Luli and Strohl [18], who showed that specific growth rate, biomass yield, and acetate formation varied significantly among different strains tested. It has also been reported that among different *E. coli* strains *E. coli* JM109 was the only strain that allowed good production of poly(L-aspartyl-L-phenylalanine) [19]. Up to now, little effort has been made to understand the physiology of P4HB synthesis in *E. coli.*

In this study, we compared P4HB production in different *E. coli* recombinants and identified the best *E. coli* strain regarding cell growth and P4HB accumulation. The effect of growth conditions in batch culture was studied for following parameters: temperature, the carbon source, and Na-4HB concentrations. Furthermore, the best physiological stage at which Na-4HB precursor should be added was investigated. P4HB productivity of 0.027 w w^-1^ h^-1^ with excellent conversion yield Y_P4HB/Na-4HB_ of 92% w w^-1^ was achieved.

## Results

### Comparison of different *E. coli* recombinants for and influence of 4HB concentrations on P4HB production

**Figure 1:**
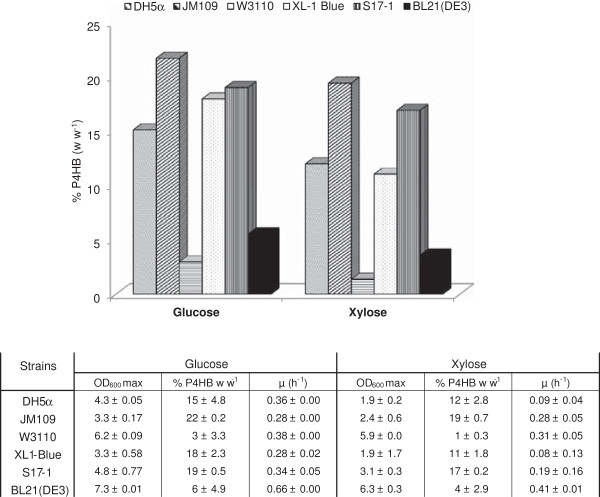
**Comparison of P4HB accumulation in six recombinant *E. coli *strains.** Cultures were grown in shake flasks at 37°C in modified E2 minimal medium containing either glucose or xylose (10 g L^-1^). Error bars represent the standard deviations from four independent measurements. There is a significant difference from t-test in the P4HB accumulation between the strains growing on glucose and on xylose with t(5) value of 3.71 and *p <* 0*.*01.

Six different *E. coli* strains were transformed with plasmid pKSSE5.3 carrying the necessary genes for P4HB synthesis, namely a PHA synthase gene (*phaC*) from *R. eutropha* and a 4-hydroxybutyric acid-coenzyme A transferase gene (*orfZ*) from *C. kluyveri*. An initial screening on the performance of the obtained recombinant strains was conducted in shake flasks containing modified E2 medium with xylose as the growth substrate and Na-4HB for P4HB synthesis. Specific growth rate, maximum optical density (OD_600_) and P4HB accumulation were measured with time. For comparison, the same experiments were performed with glucose as the growth substrate. The tested *E. coli* recombinants exhibited different specific growth rates and accumulated different amounts of P4HB (Figure 1). On both glucose and xylose, the W3110 and BL21(DE3) recombinants displayed a high specific growth rate, but accumulated only negligible amounts of P4HB. Specific growth rates of DH5α and XL1-Blue recombinants were much lower on xylose than on glucose, and the P4HB content in the range of 11% to 18% (w w^-1^) was measured during growth on both sugars. On xylose the best performer for P4HB production was the recombinant JM109, which exhibited a specific growth rate of 0.28 h^-1^ and accumulated P4HB up to 19% (w w^-1^). *E. coli* JM109 (pKSSE5.3) was thus selected for further studies.

In parallel, under the same conditions as described above and using glucose as growth substrate, the effect of the 4HB concentration on cell growth and P4HB production was investigated. When the modified E2 medium was supplemented with Na-4HB as the sole carbon source, no growth was observed for *E. coli* JM109 (pKSSE5.3), demonstrating that Na-4HB cannot be utilized by *E. coli* JM109 as carbon source for growth. The optimum concentration of Na-4HB for P4HB production was found to be between 2 g L^-1^ and 4 g L^-1^. Outside this range either low amounts of P4HB were obtained, or growth inhibition took place (Table 1). Therefore, 4 g L^-1^ Na-4HB was used in subsequent experiments.

**Table 1:** **Influence of Na-4HB concentrations on P4HB accumulation in *E. coli *** **JM109 (pKSSE5.3)**

**Na-4HB (g L**^**-1**^**)**	**OD**_**600max**_	**%P4HB (w w**^**-1**^**)**	**μ (h**^**-1**^**)**
1	1.77 ± 0.08	2 ± 0.1	0.32 ± 0.01
2	1.88 ± 0.02	21 ± 0.4	0.34 ± 0.02
4	1.94 ± 0.04	23 ± 0.7	0.33 ± 0.01
6	1.93 ± 0.06	21 ± 0.9	0.22 ± 0.01

The cells were grown at 37°C for 30 h in shake flasks containing modified E2 medium supplemented with 10 g L^-1^ glucose, 1 g L^-1^ NZ-amines and 100 μg mL^-1^ ampicillin. The standard deviations were obtained from three independent measurements.

### Comparison of carbon sources for P4HB synthesis in JM109 (pKSSE5.3)

To produce P4HB under better controlled conditions, the selected JM109 (pKSSE5.3) was cultivated in a 1 L bioreactor using modified E2 minimal medium containing xylose and 4HB. For comparison, glucose and glycerol were used as growth substrates, respectively.

Table 2 shows that the cells grown on xylose and glucose reached a similar maximal OD_600_ with a similar specific growth rate. More P4HB was produced on xylose (32% w w^-1^) than on glucose (19% w w^-1^). Grown on glycerol, the recombinant strain reached a much higher biomass than on glucose or xylose. This difference cannot be caused by P4HB accumulation because the cells synthesized only 12% (w w^-1^) of P4HB on glycerol, which is much lower than those found during growth on either glucose or xylose. This result indicates that more carbon source is channeled to biomass when grown on glycerol under the used conditions. The achieved P4HB concentration of 0.41 g L^-1^ from glycerol was also lower than that from xylose (0.65 g L^-1^ P4HB).

**Table 2:** **Comparison of carbon sources for growth and P4HB accumulation of *E. coli *** **JM109 (pKSSE5.3)**

**Carbon source**	**Xylose**	**Glucose**	**Glycerol**
OD_600_	3.4 ± 1.4	3.9 ± 1.1	7.6 ± 0.4
CDW (g L^-1^)	2.16 ± 0.37	2.04 ± 0.60	3.80 ± 0.18
μ (h^-1^)	0.32 ± 0.09	0.38 ± 0.04	0.35 ± 0.01
P4HB content% (w w^-1^)	32 ± 3.7	19 ± 6.4	12 ± 3.6
P4HB concentration (g L^-1^)	0.65 ± 0.11	0.36 ± 0.05	0.41 ± 0.00

The cells were cultivated in a 1 L bioreactors at 37°C with an agitation of 500 rpm in modified E2 minimal medium supplemented with 4 g L^-1^ Na-4HB, 1 g L^-1^ NZ-amines, 100 μg mL^-1^ ampicillin and 0.015 g L^-1^ thiamine. Xylose, glucose or glycerol was used as the growth substrate. The growth was followed with time and samples were taken after 25 h for P4HB analysis. The data were obtained from two independent cultivations.

### Influence of temperature on growth and P4HB accumulation

**Figure 2:**
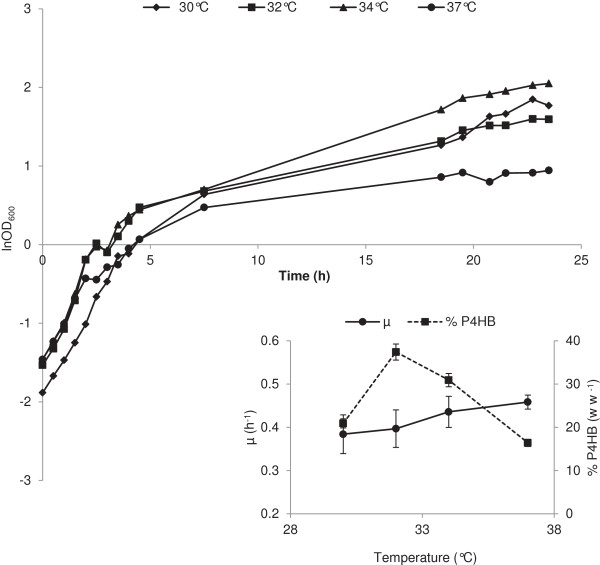
**Influence of temperature on the growth and P4HB accumulation of *E. coli *JM109 (pKSSE5.3).** The cells were grown in modified E2 minimal medium supplemented with 10 g L^-1^ xylose, 4 g L ^-1^ Na-4HB, 1 g L^-1^ NZ-amines, 100 μg mL^-1^ ampicillin and 0.015 g L^-1^ thiamine. Four different temperatures were tested: 30°C (♦), 32°C (■), 34°C (▲) and 37°C (●). Error bars represent the deviations from two independent measurements.

Optimal temperature should support cell growth as well as product formation. Therefore, the influence of temperatures at 30, 32, 34 and 37°C on growth and P4HB accumulation was investigated. As expected, with the increase of temperature the specific growth rate increased correspondingly (Figure 2). Temperature also displayed a significant impact on P4HB accumulation and the best temperature was found to be 32°C where about 37% (w w^-1^) of P4HB was produced after 24 h of cultivation. Temperatures below or above 32°C resulted in considerable decrease in P4HB content (Figure 2). Thus, cultivation temperature was set to 32°C for subsequent experiments.

### Impact of the precursor addition at different physiological growth stages on P4HB synthesis

**Figure 3:**
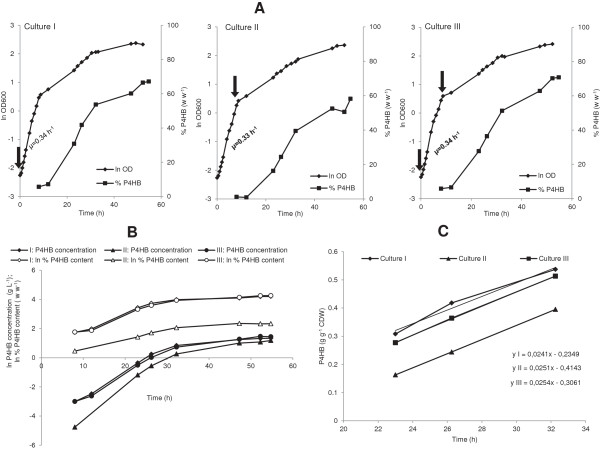
**Influence of the physiological stage of 4HB addition on P4HB synthesis. ***E. coli* JM109 (pKSSE5.3) were grown in a 1 L bioreactor at 32°C on modified E2 medium supplemented with 10 g L^-1^ xylose, 4 g L ^-1^ Na-4HB, 1 g L^-1^ NZ-amines, 100 μg mL^-1^ ampicillin and 0.015 g L^-1^ thiamine. The black arrows represent the addition of the Na-4HB precursor. Panel **A**: addition of Na-4HB at different growth stages. I: Addition of Na-4HB at the beginning of the culture; II: Addition of Na-4HB at the end of the exponential growth phase; III: Combination of addition of Na-4HB at the beginning and at the end of exponential growth phase. Panel **B**: Time courses of P4HB content and concentration presented in log-scale. Panel **C**: P4HB productivity. The P4HB accumulation rate for the described conditions is obtained from three independent cultivations.

Previously, it was found that addition of the precursor 4HB at the beginning of cultivation was best for P4HB synthesis [20]. The authors stated that addition of 4HB at the late exponential growth phase led to considerably lower cell mass reached and less P4HB accumulation due to the limited availability of CoA [20]. To investigate whether P4HB synthesis in the *E. coli* JM109 recombinant is related to cell growth (i.e., CoA availability), we conducted the following experiment: *E. coli* JM109 (pKSSE5.3) was grown on modified E2 medium containing xylose in a 1 L bioreactor at 32°C, and 4 g L^-1^ of Na-4HB was added to the culture at the beginning (culture I), at the end of the exponential phase (culture II), or by a combination of 2 g L^-1^ at the beginning and 2 g L^-1^ at the end of the exponential phase (culture III) (Figure 3A). The results obtained demonstrate that addition of 4HB at different growth phases influenced neither specific growth rate of the culture nor P4HB synthesis (Figure 3A). Cells in all cultures exhibited a specific growth rate of about 0.34 h^-1^ in the first 8 h of cultivation. In all cultures the initiation of P4HB synthesis was only at the end of the exponential growth phase, even when 4HB was provided at the beginning (cultures I and III). During the accumulation phase, P4HB content increased exponentially with a similar rate in all three cultures and to the same extent for about 24 h in all cultures (Figure 3B). Afterwards the P4HB accumulation slowed down until the end of cultivation (55 h), where the P4HB content increased to a maximum of about 70% in cultures I and III and about 60% in culture II. It seems that P4HB content in culture II could potentially increase further, however, it was not possible likely due to a limitation of certain nutrients. The concentration of P4HB (g L^-1^) increased exponentially for 18 h, starting from the initiation of P4HB synthesis at the end of the first exponential growth phase (Figure 3B). Afterwards the increase of the P4HB concentration slowed down and maximal about 3.7, 3.3 and 4.3 g L^-1^ of P4HB was obtained in cultures I, II and III, respectively, at the end of the cultivation (Figure 3B). Correspondingly, cell density in all cultures also increased exponentially with the exponential increase of P4HB synthesis (Figure 3A). The accumulation rate of P4HB per cell dry weight was linear and similar in all three cultures with a value of about 0.025 g g^-1^ h^-1^ (Figure 3C).

The results obtained here demonstrate the following: 1) P4HB synthesis only started at the end of the exponential growth phase, regardless of the stage in which the precursor 4HB was added (i.e., either at the beginning or at the end of the exponential growth phase); 2) P4HB content and concentration increased exponentially once the P4HB synthesis was initiated; 3) The P4HB accumulation rate per cell dry weight was similar regardless when the precursor 4HB was added (i.e. at the beginning or the end of the exponential growth phase); 4) The increase of biomass after the exponential growth phase was mainly due to the P4HB accumulation; and 5) P4HB accumulation stopped due to either nutritional limitation and/or product(s) inhibition. To obtain more information, a more detailed analysis on substrate consumption and product formation was performed.

### Batch culture for P4HB production

**Figure 4:**
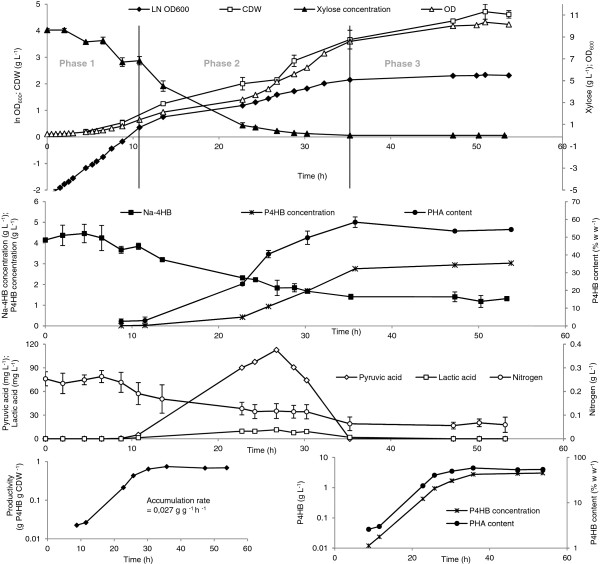
**P4HB production in batch culture in 1 L bioreactors.***E. coli* JM109 (pKSSE5.3) were grown in modified E2 medium with 10 g L^-1^ xylose as the carbon source at 32°C with an agitation of 500 rpm. The substrate consumption and product formation were followed with time. Error bars represent measurement errors of the same sample in triplicates.

*E. coli* JM109 (pKSSE5.3) was grown in a 1 L bioreactor on modified E2 medium containing xylose and Na-4HB. The cells behaved in the same manner as described above (see Figure 3) and three phases were observed (Figure 4). Phase 1: Growth phase (0–11 h). Cells grew exponentially with a specific growth rate of 0.28 h^-1^ for 11 h. In this phase, xylose and nitrogen were consumed but were still in excess in the medium. No excretion of acids such as acetic, pyruvic or lactic acid was observed during this phase. Na-4HB was hardly consumed and only a small amount of P4HB was detected (below 3% w w^-1^). The observed termination of the exponential phase can be caused either by a limited availability of nutrient(s) or by product(s) inhibition under our experimental conditions. This limitation or inhibition appears to promote P4HB synthesis. O_2_ limitation can be ruled out due to automatic control of the dissolved oxygen which was never below 30% as described in Method section. Phase 2: P4HB accumulation phase (11 – 35 h). Similar to what found before, cells started to accumulate P4HB exponentially after phase 1 and Na-4HB was consumed and decreased in the culture from 3.8 to 1.4 g L^-1^. During this time, P4HB content increased from 3% to 58% (w w^-1^) and the total amount of P4HB increased from 0.024 to 2.76 g L^-1^ (Figure 4). The residual biomass kept almost constant during this phase. Xylose and nitrogen were further consumed and the culture reached carbon (xylose) limitation after 35 h of incubation, whereas there was still enough nitrogen left. In this phase, pyruvic acid and lactic acid were produced and reached maximal concentrations of 113 mg L^-1^ and 11 mg L^-1^, respectively, after 27 h of incubation. Both acids were further consumed and depleted from the medium after 35 h of incubation. Accumulation of pyruvic acid and lactic acid cannot be the reason for the transition from Phase 1 to Phase 2 because the concentrations of both acids were too low to be inhibiting [21]. Phase 3: Stagnation phase (35 – 54 h). Upon depletion of xylose no significant change in biomass and P4HB content took place. The cells consumed neither the nitrate provided nor the Na-4HB completely. The P4HB accumulation rate was in the range of 0.027 g g^-1^ h^-1^, similar to that found in Figure 3. The consumed Na-4HB was almost completely converted into polymer with a yield Y_P4HB/Na-4HB_ of 92% g of carbon from P4HB per g of carbon from Na-4HB.

## Discussion

Despite the fact that bioprocesses for recombinant production of P3HB in *E. coli* have been studied extensively [22,23], the biosynthesis of P4HB in *E. coli* has not been yet investigated in depth. Several reports have described the P4HB synthesis and accumulation in *E. coli*[16,20,24]. However, neither physiological and cultivation conditions, nor the external factors that may influence P4HB accumulation have been studied yet in detail. For this reason, we attempted to address two issues in this work. The first issue was whether or not P4HB can be produced from Na-4HB efficiently in combination with xylose as growth substrate. The second issue was to tackle how P4HB synthesis can be stimulated. Our results demonstrate that P4HB can be synthesized efficiently by combining xylose and 4HB and its production can be enhanced reproducibly by an unknown factor, either nutrient depletion or product inhibition.

To reach efficient P4HB production, cultures exhibiting high specific growth rate, high biomass concentration and high levels of P4HB content are desired. Since the metabolic status, including the concentrations of metabolites and the rate of metabolite formation may be different from one strain to another, it is very understandable that rates of P4HB synthesis and levels of P4HB accumulation will be different from one to another. Previously, it has been reported that P3HB production can differ dramatically by using different *E. coli* strains, e.g. the wild-type *E. coli* K12 synthesized 0.4 g L^-1^ P3HB, whereas XL1-Blue produced 7.2 g L^-1^ P3HB under the same conditions [23]. In this study, we have chosen six *E. coli* strains originated from B strain (BL21(DE3)) and K12 strains including the wild type (W3110) and the K12 derivatives (DH5α, JM109, XL1-Blue, S17-1). JM109 seems to have the best physiological background for P4HB synthesis, whereas the worst performers were W3110 and BL21(DE3). The latter two strains grew fast, and used the carbon source mainly for biomass formation but produced little amount of P4HB (Figure 1).

Previously it has been reported that using *E. coli* XL1-Blue carrying pKSSE5.3, a P4HB concentration of about 4.0 g L^-1^ and P4HB content of 36% (w w^-1^) could be obtained by a fed-batch culture on M9 medium containing glucose and yeast extract and 18 g L^-1^ of 4HB [20]. The conversion yield of the precursor 4HB to P4HB (g carbon : g carbon) was about 24%. Recently, Zhou et al. reported that *E. coli* JM109 mutant carry two plasmids reached about 1.9 g L^-1^ P4HB and 35% (w w^-1^) P4HB using LB rich medium containing glucose in a batch culture [17]. Two antibiotics were needed to keep the plasmids and LB rich medium is costly. The authors also showed that in a fed-batch fermentation 7.5 g L^-1^ P4HB could be achieved by using LB medium containing a total of 90 g L^-1^ glucose after 52 hours [17]. The conversion yield of the precursor glucose to P4HB (g carbon : g carbon) was about 10.5%. In the current study, we achieved 4.3 g L^-1^ P4HB and 67% (w w^-1^) P4HB in a batch culture using the described medium. The consumption of the precursor 4HB was almost complete with a conversion yield Y_P4HB/Na-4HB_ of 92% g g ^-1^. Even though the cost of 4HB is higher than glucose, the price of 4HB can be significantly reduced by using gamma-butyrolactone as the precursor for chemical synthesis of 4HB (see Methods section). Hence, the process developed here is an efficient approach for P4HB production.

In earlier studies, addition of 4HB at the beginning of a cultivation was found to be the best for cell growth and P4HB production [20]. Here, we observed no difference in cell growth and P4HB synthesis between adding 4HB at the beginning and at the end of the exponential growth phase (Figure 3). P4HB synthesis was initiated only at the end of exponential growth, even when 4HB was supplied right at the start. In contrast to P3HB accumulation in *E. coli,* where the polymer is synthesized during cell growth [25], P4HB production has been found to be distinctly separated from exponential cell growth in our experiments. The end of exponential growth caused by either product inhibition or nutrient limitation stimulated P4HB synthesis. It seems that the cell growth and P4HB production compete with each other for the same nutrients. As indicated from the results shown in Figure 1, both W3110 and BL21(DE3) strains grew fast and reached high final biomass but accumulated only a negligible amount of P4HB. Furthermore, when the conditions are favored for cell growth e.g. at 37°C, P4HB is disadvantaged (Figure 2). These results suggest that nutrients are directed mainly into the tricarboxylic acid (TCA) cycle for cell growth rather than into P4HB synthesizing pathway. We also did not observe the accumulation of acetic acid during the whole cultivation period. This seems to be due to the efficient utilization of excessive acetyl-CoA for the synthesis of P4HB, which would otherwise form acetic acid [26].

**Figure 5:**
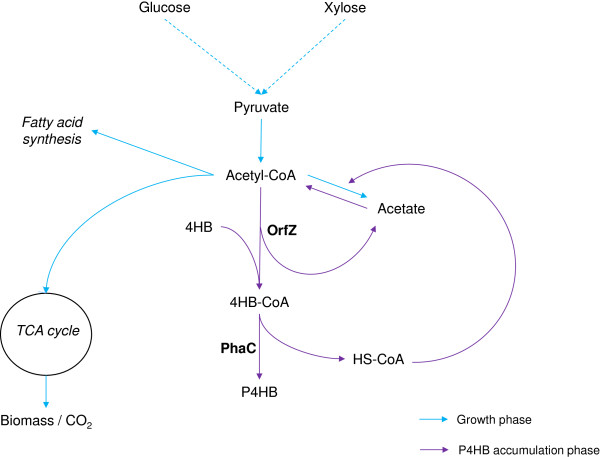
**Hypothetic metabolic pathway of P4HB synthesis from Na-4HB in recombinant *E. coli.*** Blue color represents growth phase, purple color represents P4HB synthesis phase.

Taking advantage of the knowledge acquired previously and our findings in this study, we propose a model to explain the metabolism of P4HB synthesis in recombinant *E. coli*: Introducing PHA synthase (PhaC) from *R. eutropha* and 4-hydroxybutyrate CoA-transferase (OrfZ) from *C. kluyveri* into *E. coli* would result in the establishment of a new metabolic pathway, which competes with several existing pathways leading to citrate and acetate formation and to fatty acid synthesis (Figure 5). When the available nutrients and energy are used for cell growth, P4HB would hardly be synthesized. When the cell growth slows down / stops due to nutrient limitation other than carbon starvation, P4HB synthesis can then be initiated. The reduction or stop of cell growth cannot be caused by carbon limitation because the cells still need the essential nutrients for maintenance. When xylose limitation occurred, P4HB synthesis also terminated (Figure 4).

## Conclusions

In this study, we compared for the first time different *E. coli* strains with respect to growth on xylose and P4HB accumulation. Different growth conditions were also investigated such as temperature and the cell physiological stages for P4HB synthesis. Unlike what has been reported previously, the P4HB synthesis was found to be separated from the cell growth, namely P4HB synthesis mainly takes place after the end of the exponential growth phase. Under the tested conditions, P4HB contents in the range of 58 to 70% (w w^-1^) and P4HB concentrations in the range of 2.8 to 4.3 g L^-1^ were obtained with a conversion yield Y_P4HB/Na4HB_ of 92% w w^-1^. These results were achieved here by simple batch culture, which was only possible previously through fed-batch high cell density cultures. However, to further improve the productivity of P4HB production process for practical industrial applications, high cell density cultures will need to be investigated and employed.

## Methods

### Bacterial strains and plasmids

The *E. coli* strains used in this study are listed in Table 3. Among them, XL1-Blue, S17-1 and JM109 were previously used for P4HB production [16,20,24], and thus were selected here for comparison purpose. The previously constructed plasmid pKSSE5.3 carrying a PHA synthase gene (*phaC*) from *R. eutropha* and a 4-hydroxybutyric acid-coenzyme A transferase gene (*orfZ*) from *C. kluyveri* was used in this study [16].

**Table 3:** ***E. coli *** **strains and plasmid used in this study**

**Strains**	**Relevant characteristics**	**References**
DH5α	*F*^–^, ø80d*lacZ*ΔM15, Δ(*lacZYA-argF*)U169, *deo*R, *recA*1, *endA*1, *hsdR*17(rK^–^, mK^+^), *gln*V44, *supE*44, λ^-^, *thi*-1, *gyrA*96, *relA*1, *nup*G	[27]
JM109	*endA*1, *glnV*44, *thi*-1, *relA*1, *gyrA*96, *recA*1, *mcrB*^+^, Δ(*lac-proAB*), e14-, [F’ *traD*36, *proAB*^+^, *lacI*^q^, *lacZ*ΔM15], *hsdR*17(r_K_^-^m_K_^+^)	[28]
XL-1 Blue	*endA*1, *gyrA*96(*nal*^R^), *thi*-1, *recA*1, *relA*1, *lac*, *glnV*44, F’[ ::Tn10, *proAB*^+^, *lacI*^q^, Δ(*lacZ*)M15], *hsdR*17(r_K_^-^ m_K_^+^)	[29]
S17-1	*tmpR, spcR, strR, recA pro hsdR RP4-2-Tc::Mu-Km::Tn7*	[30]
W3110	*F,*^*-*^*λ*^*-*^*, rph*-1*, INV(rrnD, rrnE)*	[31]
BL21(DE3)	*F*^*-*^*, ompT, gal, dcm, lon, hsdS*_*B*_*(r*_*B*_^*-*^*m*_*B*_^*-*^*), λ(DE3), [lacI lacUV5-T7 gene 1 ind1 sam7 nin5])*	[32]
Plasmid		
pKSSE5.3	*phaC, orfZ, Ampr*	[16]

### Chemicals, media and cultivation conditions

#### Chemicals

All chemicals were purchased from Sigma-Aldrich (Buchs, Switzerland).

#### Synthesis of Na-4HB

One of the simplest and low-cost ways to obtain 4HB is by hydrolysis of the corresponding lactone to the desired hydroxy acid. The reaction was proceeded with equal molar of gamma-butyrolactone and NaOH [33]. In detail: 4 M NaOH solution was prepared and mixed slowly to 4 M of gamma-butyrolactone on ice. After the reaction mixture was cooled down to room temperature, it was analyzed by HPLC/MS ([34], also see below). An almost 100% conversion of gamma-butyrolactone to Na-4HB was achieved.

Gamma-butyrolactone + NaOH → Na-4HB.

#### Media

*E. coli* strains were cultivated overnight in LB medium with 100 μg mL^-1^ ampicillin. This culture was used to inoculate the preculture containing modified E2 medium [35]. Modified E2 medium contained the following constituents: NaNH_4_HPO_4_ · 4H_2_O 3.5 g L^-1^, KH_2_PO_4_ 3.7 g L^-1^, K_2_HPO_4_ 7.5 g L^-1^, dissolved in 1 L of water. One mL L^-1^ of 1 M MgSO_4_ · 7H_2_O was added to the medium. One mL L^-1^ of trace elements (TE) dissolved in 1 M HCl was also added. TE contained: FeSO_4_ · 7H_2_O 2.78 g L^-1^, CaCl_2_ · 2H_2_O 1.47 g L^-1^, MnCl_2_ · 4H_2_O 1.98 g L^-1^, CoCl_2_ · 6H_2_O 2.38 g L^-1^, CuCl_2_ · 2H_2_O 0.17 g L^-1^, ZnSO_4_ · 7H_2_O 0.29 g L^-1^. Xylose, glucose or glycerol (10 g L^-1^) was used as the sole carbon source.

#### Growth in shake flasks

Growth studies were performed in 1 L shake flasks containing 200 mL of modified E2 medium and 10 g L^-1^ of a carbon source. 1 g L^-1^ of NZ-amines and 100 μg mL^-1^ of ampicillin were added to the minimal medium. Na-4HB (1 to 6 g L^-1^ according to the experiment) was added as P4HB precursor as indicated in individual experiments.

#### Culture in 1 L bioreactors

Four 1 L reactor cultures were grown in parallel in Multifors benchtop bioreactors (Infors AG, Bottmingen, Switzerland). Temperature was controlled at 32°C with an external circulating water bath, and pH was maintained at 7.0 +/− 0.1 by automatic addition of 25% NaOH or 30% H_3_PO_4_. Dissolved oxygen tension was monitored continuously with an oxygen probe (Infors AG, Bottmingen, Switzerland) and kept always above 30% oxygen saturation. The agitation was set at 500 rpm. Each reactor was inoculated using a preculture prepared as described above in “Growth in shake flasks”. The initial OD_600_ value in bioreactors was between 0.10 and 0.30. The modified E2 medium was used to perform all the growth studies in 1 L reactors supplemented with 10 g L^-1^ of carbon source, 1 g L^-1^ of NZ-amines, 4 g L^-1^ of Na-4HB and 0.015 g L^-1^ of thiamine. Ampicillin was added to a final concentration of 100 μg mL^-1^ to maintain the pKSSE5.3 plasmid.

### Analytical methods

#### Cell concentration

Growth of bacterial cells was followed by measuring optical density at 600 nm (OD_600_) using a UV spectrophotometer (Genesys 6, ThermoSpectronic, Switzerland).

Cell dry weight was determined either by using pre-weighed polycarbonate filters (pore size 0.2 μm, Whatman, Scheicher & Schuell, Dassel, Germany) or by pre-weighed 2 mL Eppendorf tubes. In the first method, an appropriate volume (0.5 to 5 mL) of culture was filtered in order to obtain a biomass dry weight of about 2 mg per filter. The filter was dried overnight at 100°C, cooled down to room temperature in a desiccator and then weighed. In the second method, 2 mL of culture broth was centrifuged at 12,000 g for 2 min in a 2 mL pre-weighed Eppendorf tube. The supernatant was discarded and the cell pellet was dried overnight at 100°C and cooled down to room temperature in a desiccator. The 2 mL Eppendorf tube was then weighed. For both methods, the weight difference was used to determine the dry biomass.

#### PHA content

PHA content and composition were determined according to a method described previously [36]. Methylene chloride containing benzoic acid (0.1 g L^-1^) was used as internal standard. Own lab purified P4HB was used for obtaining standard curves. Na_2_CO_3_ powder was added to dry the extracted chlorinated solvent phase. The samples were analyzed by gas chromatography (GC) (A200s, Trace GC 2000 series, Fisons Instruments, Rodano, Italy) equipped with a polar fused silica capillary column (Supelcowax-10: length 30 m; inside diameter 0.31 mm; film thickness 0.5 μm; Supelco, Buchs, Switzerland) [37]. P4HB was depolymerized, esterified and methylated, leading to three different peaks in the GC chromatogram. These three peaks were also observed by Hein and coworkers when P4HB homopolymers were analyzed [16].

#### Nitrogen concentration

NH_4_^+^-nitrogen consumption was detected using an ammonium test kit following the manufacturer instruction (Merck KGaA, 64271 Darmstadt, Germany). The detection limit was 0.01 NH_4_^+^-nitrogen mg L^-1^. The method was linear up to 3.0 mg L^-1^, above which dilution with distilled water was needed. The results obtained are in mg L ^-1^ of nitrogen.

#### Measurement of xylose, Na-4HB, acetate, pyruvate and lactate

Concentrations of xylose, Na-4HB and acetate in the culture medium were measured by HPLC/MS. Supernatant resulting from culture centrifugation at 12,000 g for 2 min was diluted to a concentration between 0.01 and 0.1 mM with distilled water, filtrated through a Titan HPLC filter (0.45 μm, Infochroma AG, Zug, Switzerland), and loaded on a reversed phase C18 column (Gemini C18 5 micron, 250 mm × 4.60 mm, Phenomenex, U.K.). A gradient of 100% of diluted formic acid (0.1 v% in water) to 100% of acetonitrile was applied as the mobile phase. The flow rate was 0.8 mL min^-1^ and the gradient was completed after 25 minutes. The peaks were detected by electrospray ionization (ESI) in negative mode [34]. Standard curves for xylose, Na-4HB and acetate were recorded in the range of 0.01 to 1.00 g L^-1^, 0.01 g L^-1^ to 0.20 g L^-1^ and 0.01 to 1.00 g L^-1^, respectively.

Pyruvate and lactate in the culture supernatant were measured by ion chromatography (IC) (Metrosep A SUPP 5 250, 4 × 250 mm). A flow of 0.7 mL min^-1^ of eluent containing 1 mM NaHCO_3_ was used. Both acids were detected using a conductivity detector. A volume of 20 μL of sample diluted with water to a range of 50 to 250 ppm was injected and analyzed by IC system. Pure pyruvate and lactate were used to generate standard curves.

### Calculation of conversion rate

Consumed Na-4HB was determined by the difference between the Na-4HB amount supplied at the beginning of a cultivation and Na-4HB content left over in the medium after the cultivation. The concentration of P4HB (g L^-1^) was determined from cell dry weight (CDW) in g L^-1^ and the cellular content of P4HB (w w^-1^) obtained at the end the cultivation. The conversion rate was calculated by dividing the mass of carbon in gram from P4HB with the mass of carbon in gram from Na-4HB (w w^-1^).

## Competing interests

The authors declare that they have no competing interest.

## Authors’ contributions

SLM designed and performed the experiments, prepared and revised the manuscript. MZ and TE participated in designing the experiment and in revising the final manuscript. LTM revised the final manuscript. QR designed and supervised the experiments, prepared and revised the manuscript. All authors read and approved the final manuscript.
